# Risk scores for predicting early antiretroviral therapy mortality in sub-Saharan Africa to inform who needs intensification of care: a derivation and external validation cohort study

**DOI:** 10.1186/s12916-020-01775-8

**Published:** 2020-11-09

**Authors:** Andrew F. Auld, Katherine Fielding, Tefera Agizew, Alice Maida, Anikie Mathoma, Rosanna Boyd, Anand Date, Sherri L. Pals, George Bicego, Yuliang Liu, Ray W. Shiraishi, Peter Ehrenkranz, Christopher Serumola, Unami Mathebula, Heather Alexander, Salome Charalambous, Courtney Emerson, Goabaone Rankgoane-Pono, Pontsho Pono, Alyssa Finlay, James C. Shepherd, Charles Holmes, Tedd V. Ellerbrock, Alison D. Grant

**Affiliations:** 1 Division of Global HIV & TB, United States Centers for Disease Control and Prevention (CDC), Nico House, City Centre, P.O. Box 30016, Lilongwe 3, Malawi; 2 TB Centre, London Sch. of Hygiene & Tropical Med, London, UK; 3 School of Public Health, University of the Witwatersrand, Johannesburg, South Africa; 4 Division of TB Elimination, Centers for Disease Control and Prevention, Gaborone, Botswana; 5 Division of Global HIV & TB, Centers for Disease Control and Prevention, Atlanta, GA USA; 6 Bill and Melinda Gates Foundation, Seattle, WA USA; 7 Aurum Institute, Johannesburg, South Africa; 8 Ministry of Health and Wellness, Gaborone, Botswana; 9 Yale University School of Medicine, New Haven, CT USA; 10 Center for Global Health Practice and Impact, Georgetown University Medical Center, Washington D.C, USA; 11 Africa Health Research Institute, School of Nursing and Public Heath, University of KwaZulu-Natal, Durban, South Africa

**Keywords:** HIV, Acquired immuno-deficiency syndrome, Antiretroviral therapy, Mortality, Predictive models, Clinical scores

## Abstract

**Background:**

Clinical scores to determine early (6-month) antiretroviral therapy (ART) mortality risk have not been developed for sub-Saharan Africa (SSA), home to 70% of people living with HIV. In the absence of validated scores, WHO eligibility criteria (EC) for ART care intensification are CD4 < 200/μL or WHO stage III/IV.

**Methods:**

We used Botswana XPRES trial data for adult ART enrollees to develop CD4-independent and CD4-dependent multivariable prognostic models for 6-month mortality. Scores were derived by rescaling coefficients. Scores were developed using the first 50% of XPRES ART enrollees, and their accuracy validated internally and externally using South African TB Fast Track (TBFT) trial data. Predictive accuracy was compared between scores and WHO EC.

**Results:**

Among 5553 XPRES enrollees, 2838 were included in the derivation dataset; 68% were female and 83 (3%) died by 6 months. Among 1077 TBFT ART enrollees, 55% were female and 6% died by 6 months. Factors predictive of 6-month mortality in the derivation dataset at *p* < 0.01 and selected for the CD4-independent score included male gender (2 points), ≥ 1 WHO tuberculosis symptom (2 points), WHO stage III/IV (2 points), severe anemia (hemoglobin < 8 g/dL) (3 points), and temperature > 37.5 °C (2 points). The same variables plus CD4 < 200/μL (1 point) were included in the CD4-dependent score. Among XPRES enrollees, a CD4-independent score of ≥ 4 would provide 86% sensitivity and 66% specificity, whereas WHO EC would provide 83% sensitivity and 58% specificity. If WHO stage alone was used, sensitivity was 48% and specificity 89%. Among TBFT enrollees, the CD4-independent score of ≥ 4 would provide 95% sensitivity and 27% specificity, whereas WHO EC would provide 100% sensitivity but 0% specificity. Accuracy was similar between CD4-independent and CD4-dependent scores. Categorizing CD4-independent scores into low (< 4), moderate (4–6), and high risk (≥ 7) gave 6-month mortality of 1%, 4%, and 17% for XPRES and 1%, 5%, and 30% for TBFT enrollees.

**Conclusions:**

Sensitivity of the CD4-independent score was nearly twice that of WHO stage in predicting 6-month mortality and could be used in settings lacking CD4 testing to inform ART care intensification. The CD4-dependent score improved specificity versus WHO EC. Both scores should be considered for scale-up in SSA.

## Background

Over the last 16 years, the scale-up of HIV treatment globally has reached over 24.5 million people living with HIV (PLHIV) with lifesaving antiretroviral therapy (ART), resulting in declines in both HIV-associated mortality and HIV incidence [1–3]. However, each year there are still about 770,000 global AIDS-related deaths, with 470,000 (61%) of these deaths occurring in sub-Saharan Africa (SSA) [1]. To reduce AIDS-related mortality, the global community is striving to reach 2030 targets of ensuring at least 90% of PLHIV are on ART [4], which will require ART enrollment for an additional 10 million of the 37.9 million PLHIV globally, about two-thirds of whom live in sub-Saharan Africa (SSA) [1]. Mortality rates during ART are highest in the first 6 months of therapy, and these early ART mortality rates continue to be highest in SSA [5, 6]. If 2030 goals of reducing AIDS-related mortality by 90% compared with 2010 are to be met, substantial progress needs to be made in addressing early ART mortality in SSA [5, 6], where 20–40% of new ART enrollees still initiate ART with relatively advanced HIV disease [7, 8].

To achieve these mortality reductions, efficient use of available resources through differentiated service delivery (DSD) models to provide tailored, patient-centered care will be needed [9, 10]. The World Health Organization (WHO) currently recommends intensification of care for persons > 5 years old starting ART with advanced HIV disease as defined by CD4^+^ T cell (CD4) count < 200 cells/μL or WHO stage III/IV [8]. The intensification of care package, which has been shown to reduce early mortality [11], includes cotrimoxazole prophylaxis, tuberculosis (TB) screening with subsequent TB treatment or TB preventive therapy, cryptococcal antigen (CrAg) screening with pre-emptive therapy for eligible CrAg-positive people, and enhanced adherence counseling. However, the majority of health facilities providing ART in low- and middle-income countries (LMIC) lack access to rapid or point-of-care (POC) CD4 testing [8]. In these settings, up to half of adults with a CD4 count < 100/μL could be categorized as WHO stage I/II and would be missed by an advanced disease screening algorithm that relied on WHO stage alone [11]. In addition, a screening tool for advanced disease that relies only on CD4 count and WHO disease stage misses the many other demographic and clinical predictors associated with early ART mortality [9]. To date, most analyses evaluating eligibility for DSD models have focused on identifying stable patients for de-escalation of care [9]. Only one analysis from Haiti has evaluated a clinical score for determining who needs intensification of early ART care, and this was not externally validated [12].

Therefore, we evaluated whether a clinical score derived from easily available covariates at ART initiation in resource-constrained clinic settings could better predict who is at risk for early (6-month) ART mortality than the current WHO advanced disease eligibility criteria. We developed clinical scores to help predict early ART mortality risk for two scenarios: (1) a scenario where on-site/rapid off-site CD4 testing is not available as is the case for the majority of ART clinics in LMIC and (2) a scenario where on-site/rapid off-site CD4 testing is available.

## Methods

We used data from the Xpert Package Rollout Evaluation using a Stepped-wedge design (XPRES) trial to derive the two clinical scores to help clinicians identify those at the highest risk of early ART mortality and therefore in need of ART care intensification [13]. The first clinical score assumes CD4 is unavailable at ART initiation (i.e., a CD4-independent score) and the second clinical score assumes CD4 count is available (i.e., a CD4-dependent score). We used the first 50% of XPRES cohort enrollees to derive a prediction model, and the second 50% to internally validate the model. We then used data from the TB Fast Track (TBFT) trial in South Africa (SA) to externally validate the derived clinical scores [14]. We compared the screening accuracy of our derived clinical scores with existing CD4-based WHO eligibility criteria for advanced disease and ART care intensification.

### XPRES study design and participants for prediction tool development

XPRES was a multi-center, stepped-wedge cluster randomized trial with a retrospective baseline component conducted at 22 health facilities, including five hospitals and 17 clinics, that were purposively selected to be representative of HIV treatment clinics in Botswana [13]. In the prospective, stepped-wedge portion of the trial, all non-incarcerated, consenting, ART-naïve, HIV-positive persons, regardless of TB treatment or symptom status, presenting to the study clinics between August 2012 and end of March 2014, were eligible for enrollment. Only adolescents and adults (aged ≥ 12 years old) were included in this analysis.

### XPRES procedures

Per Botswana national guidelines during the time period of the study, all XPRES study participants were eligible for ART initiation if they had a CD4 count ≤ 350 cells/μL, were diagnosed as having WHO stage III/IV events, or were pregnant or breastfeeding [15]. All study participants received clinical care and follow-up appointments per Ministry of Health (MOH) guidelines (see Additional file 1, a table summarizing standard clinical care follow-up).

#### Interventions

The prospective XPRES cohort was recruited within two phases of the stepped-wedge trial. In the first phase, all prospective XPRES participants received two enhanced care interventions in addition to standard of care: (1) additional support for intensified TB case finding and (2) intensified tracing for patients missing clinic appointments. In the second phase, the Xpert® MTB/RIF assay (Cepheid; Sunnyvale, CA) (Xpert) was initiated in place of sputum smear microscopy for TB diagnosis. Details of these interventions have been previously published [16] and are provided in a supplementary appendix (see Additional file 2, text describing enrollment and enhanced care interventions). We have previously shown that there was no significant difference in 6-month ART mortality between the two prospective phases of XPRES [16]. Enrollment and follow-up procedures are described in the supplementary appendix (see Additional file 2, text). XPRES participants were followed for 12 months, or until the end of TB treatment, whichever was later. The final follow-up visits for XPRES enrollees were in June 2015.

### Development and temporal validation of the prediction model

A clinically useful prediction model should demonstrate accurate prediction of the outcome in data other than that in which the model was developed. Therefore, we split the XPRES dataset in a 1:1 ratio using the mid-point of enrollment at each of the 22 study clinics to create the derivation dataset (the first 50% of enrollees) and the temporal validation dataset (the second 50% of enrollees) [17].

#### Outcome

The outcome of interest for both the XPRES trial and this analysis was early (6-month) ART mortality. We implemented intensive efforts to ascertain true mortality outcomes among participants, with deaths and date of death either passively reported to the clinic by friends or relatives or actively ascertained if the client had missed an appointment or was considered lost to follow-up (LTFU) (> 60 days late for a scheduled appointment) [18]. Initial efforts to ascertain outcomes of clients who missed an appointment by ≥ 1 day included up to five phone calls to the client or contact and up to two home visits. In addition, for all clients unreachable by phone or home visit who met the LTFU definition, vital status was ascertained through national Death Registry review. By law, since 1969, all deaths need to be registered in the Death Registry, which is maintained by the Botswana Civil and National Registration Office. Available data shows Death Registry data completeness to be high [16].

#### Candidate predictor variables

We selected candidate predictor variables for potential inclusion in the predictive model based on prior publications, and the need for variables to be reproducible, objective, and readily available in resource-constrained clinic settings [19]. We considered variables known to be associated with mortality including age, sex (coded as male, pregnant female, and non-pregnant female [20]), education level, employment status, smoking history, prior TB treatment, number of WHO TB symptoms, weight, body mass index (BMI) (weight/height^2^), hemoglobin level, CD4 count, temperature at ART initiation in degrees Celsius, and respiratory rate at ART initiation [20–23].

Within the derivation dataset, we performed univariable analyses assessing the association of each variable with risk of mortality using logistic regression. Because follow-up of all XPRES and TBFT enrollees was complete with true ascertainment of 6-month mortality outcomes, 6-month risk was preferred to rate [16]. Continuous variables were assessed for non-linearity with log odds of death using fractional polynomials, as well as by comparing Akaike’s Information Criteria and Bayesian Information Criteria between models with linear or fractional polynomial terms. Where non-linearity was observed, the appropriate fractional polynomial terms were included in the logistic regression. We also examined scatter plots of linear and transformed continuous variables and risk of mortality to assess inflection points which might inform appropriate categorization of continuous variables.

For the multivariable analysis, a complete case analysis, whereby observations with missing data for key variables were dropped, was chosen because few data (*<* 10%) were missing. To generate a parsimonious multivariable model, we used a stepwise backward elimination approach, starting with all candidate variables and excluding variables sequentially if *p >* 0.01 using both automatic and manual approaches. We also explored how findings changed using a forward stepwise addition approach. Where two or more predictors were highly correlated, only one was selected, to simplify the prognostic model. We created two multivariable models: one in which CD4 was purposefully excluded and one in which CD4 count was included as a candidate variable to reflect situations where CD4 is either unavailable or available at the clinic. Plausible interactions between covariates (e.g., between CD4 and age) were assessed using the likelihood ratio test.

In both the derivation and temporal validation datasets, we assessed multivariable model calibration (i.e., the agreement between probability of 6-month mortality predicted by the model and observed probability of death within quantiles of predicted risk) graphically in a calibration plot [17] and statistically using the Hosmer-Lemeshow test. We also assessed discrimination, the ability of our model to differentiate patients who died by 6 months of ART vs. those who did not, using the area under the receiver-operating characteristic (AUROC) curve, also referred to as the C-statistic or C-index. AUROC values of 0.7 to 0.79, 0.8 to 0.89, and ≥ 0.9 are respectively considered acceptable, excellent, and outstanding discrimination [24].

Two final multivariable models were used to generate the two clinical scores (i.e., the CD4-independent and CD4-dependent scores). For these models, continuous variables were categorized in a clinically meaningful manner based on their functional form and information from the published literature. Each beta coefficient from this logistic regression model was then rescaled to generate a clinical score by dividing each coefficient by the smallest positive model coefficient and rounding to the nearest integer. The total number of points was summed for each participant to calculate their total clinical score.

### External validation of risk scores

To externally validate the clinical risk score, we used data collected independently from the TBFT trial from SA [14]. TBFT was an open-label cluster randomized controlled trial, recruiting individuals from 24 primary healthcare clinics in SA. All outpatient, HIV-positive adults (aged ≥ 18 years) with CD4 counts < 150/μL, no TB treatment in the past 3 months, and no ART in the last 6 months were eligible. In the intervention clinics, participants were classified by a study algorithm as having high, medium, or low TB risk. High TB risk patients (i.e., those with positive lateral flow urine lipoarabinomannan assay [LF-LAM], BMI < 18.5, or hemoglobin < 10 g/dL) started TB treatment immediately followed by ART 2 weeks later. Medium TB risk participants (i.e., those with ≥ 1 WHO TB symptom only) were recommended to have symptom-guided TB investigation. Low TB risk patients (no TB symptoms or high-risk criteria) were recommended to start ART immediately. The primary outcome was all-cause mortality at 6 months after enrollment. We restricted this analysis to intervention arm participants, for whom key variables such as temperature at enrollment were available, and to those patients who started ART, since the outcome of interest was mortality within the first 6 months of ART. The median time from trial enrollment to ART start in the intervention arm was 21 days. Participants were enrolled in TBFT between December 19, 2012, and December 18, 2014. The clinical risk score for mortality was calculated by assigning the same “points” to variables as for the derivation cohort.

For both the XPRES cohort (combined derivation and validation datasets), and the TBFT datasets, we explored how sensitivity, specificity, positive predictive value (PPV), negative predictive value (NPV), and AUROC curve values varied with increasing clinical score in terms of predicting 6-month mortality and compared this screening accuracy and discrimination performance with the WHO eligibility criteria for advanced disease. Three risk groups were created to visualize increasing 6-month ART mortality risk with increasing clinical score, and the percentage of ART enrollees falling into each risk group. Kaplan-Meier (K-M) curves were used to visualize rates of early mortality within the three risk groups.

All analyses were conducted using STATA 16 (StataCorp, 2009, Stata Statistical Software, Release 16, College Station, TX). The study is reported in concordance with TRIPOD guidance for multivariable prediction models (see Additional file 3, a table with the TRIPOD checklist).

## Results

**Fig. 1:**
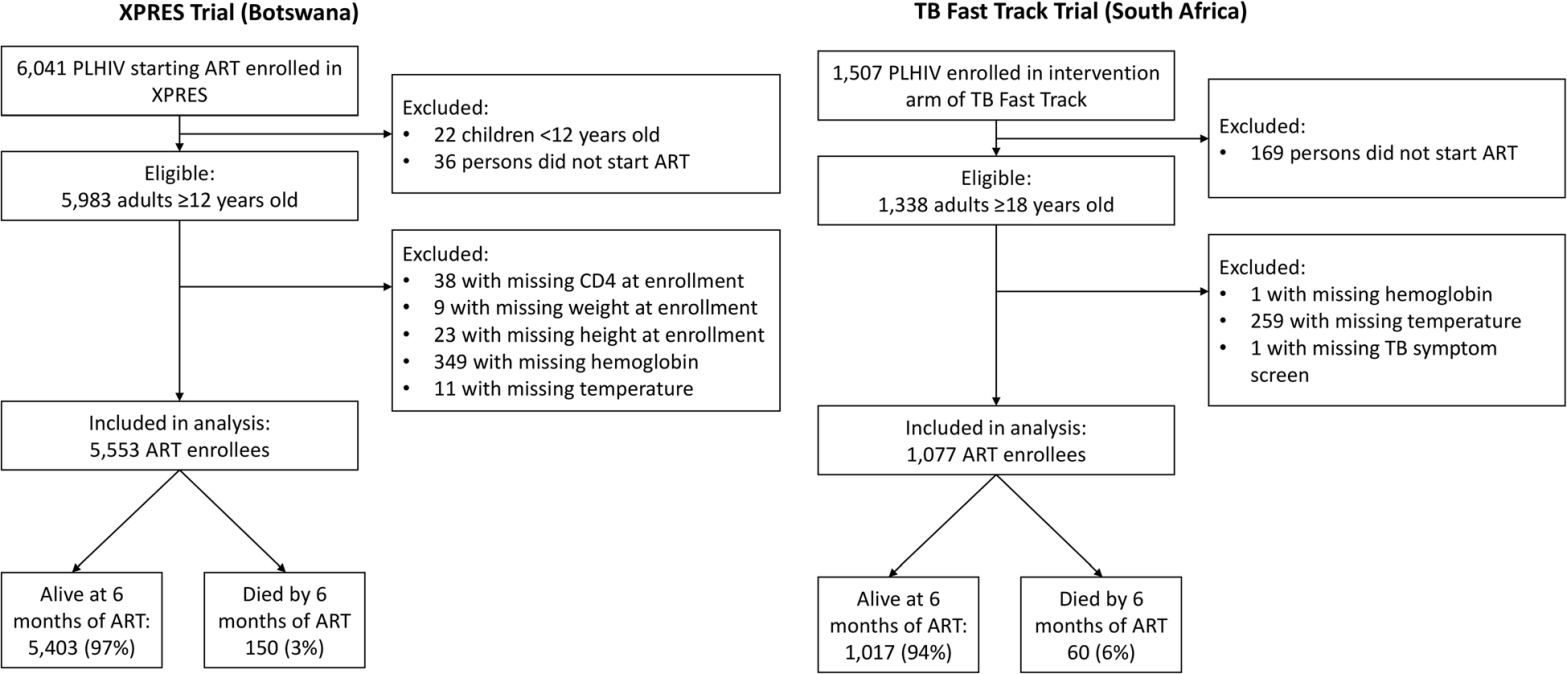
Study profile

From the XPRES cohort, 5553 eligible ART enrollees with complete data for candidate predictors were included in the analysis (Fig. 1). Overall, 150 (3%) of 5553 ART enrollees died within 6 months of ART initiation.

### Internal derivation and temporal validation datasets

From the XPRES cohort, the internal derivation (*N* = 2838) and temporal validation (*N* = 2715) datasets were created (Table 1). Key characteristics including median age (34), percentage female (66–68%), median CD4 (240–245/μL), and 6-month mortality (2.5–2.9%) were similar between internal XPRES derivation and validation datasets (Table 1). Notable differences between the XPRES cohorts and TBFT external validation cohort were that the TBFT cohort had a higher prevalence of markers of advanced disease, with a higher prevalence of ≥ 1 TB symptom (79% versus 30%), lower median CD4 count (72 versus 240–245/μL), and higher incidence of all-cause 6-month ART mortality (6.0% versus 2.5–2.9%) (Table 1).

**Table 1:** Comparison of characteristics of antiretroviral therapy enrollees between internal derivation, internal validation, and external validation datasets

		Internal derivation dataset (***N*** = 2838)		Internal validation dataset (***N*** = 2715)		External validation dataset (TB Fast Track, SA; ***N*** = 1077)^a^	
Demographics		*n*	Median or %	*n*	Median or %	*n*	Median or %
Age, years, median (IQR)		2838	33.8 (28.6–40.9)	2715	34.0 (28.6–41.4)	1077	38.0 (32.0–44.0)
Female, *n*, %		1938	68%	1779	66%	590	55%
If female, pregnant, *n*, %		520	27%	551	31%	0	0%
Marital status, *n*, %	Married/civil union	300	11%	265	10%		
	Single	2441	86%	2346	86%		
	Widowed/divorced	97	3%	104	4%		
Smoking history (ever smoked), *n*, %		517	18%	551	20%	238	22%
Currently employed, *n*, %		1270	45%	1286	47%		
Education, *n*, %	None	196	7%	200	7%		
	Primary	687	24%	596	22%		
	Secondary	1734	61%	1641	60%		
	Higher	221	8%	278	10%		
**HIV/TB history**							
Previous TB treatment, *n*, %	Yes	277	10%	262	10%		
WHO TB symptoms, *n*, %							
Cough	Yes	495	17%	547	20%	463	43%
Weight loss	Yes	599	21%	555	20%	797	74%
Fever	Yes	259	9%	245	9%	314	29%
Night sweats	Yes	273	10%	253	9%	348	32%
Number of WHO TB symptoms, *n*, %	0	1975	70%	1911	70%	230	21%
	1	427	15%	349	13%	285	26%
	2	202	7%	216	8%	226	21%
	3	141	5%	137	5%	178	17%
	4	93	3%	102	4%	158	15%
**Clinical characteristics**							
WHO stage III/IV, *n*, %		354	12%	307	11%		
CD4 (cells/μL)	Median (IQR)	2838	245 (143–315)	2715	240 (134–319)	1077	72 (36–110)
Weight (kilograms)	Median (IQR)	2838	58.8 (51.8–68.2)	2715	59.7 (52.8–69.0)	1077	57.9 (50.8–67.0)
BMI (weight/height²)	Median (IQR)	2838	21.5 (18.9–24.9)	2715	21.6 (19.1–25.0)	1077	21.3 (18.8–25.0)
Hemoglobin (g/dL)	Median (IQR)	2838	11.9 (10.4–13.2)	2715	12.0 (10.7–13.4)	1077	11.3 (9.7–13.0)
Temperature (degrees Celsius)	Median (IQR)	2838	36.2 (35.8–36.5)	2715	36.2 (35.8–36.6)	1077	36.4 (36.0–36.7)
Respiratory rate (breaths/min)	Median (IQR)	2838	19 (18–20)	2715	19 (18–20)		
Heart rate (beats/min)	Median (IQR)	2838	84 (75–94)	2715	84 (75–95)		
**Mortality within 6 months**							
Cumulative incidence, *n*, %		83	2.9%	67	2.5%	60	6%
Time to death (days)	Median (IQR)	83	50 (25–105)	67	46 (16–87)	60	55 (30–112)

*Abbreviations*: *IQR* interquartile range, *TB* tuberculosis, *WHO* World Health Organization, *CD4* CD4^+^ T cell count, *TB* tuberculosis, *BMI* body mass index, *SA* South Africa

^a^TBFT study enrollees in the intervention arm who started ART

### Development of the regression model

Table 2 summarizes the results of univariable and multivariable logistic regression model development. Although the linear continuous variables of age, weight, BMI, heart rate, and respiratory rate, as well as history of smoking, were associated with 6-month mortality in univariable analysis, these variables were either dropped as candidate variables due to correlation (i.e., weight and heart rate were dropped due to correlation with BMI and temperature respectively) or eliminated in the stepwise backward elimination approach due to *p* values in multivariable analysis > 0.01.

**Table 2:** Univariable and multivariable logistic regression analysis in the derivation dataset (*N* = 2838)

		Alive/TF by 6 months of ART (***N*** = 2755)			Died by 6 months of ART (***N*** = 83)			Unadjusted			Model A—adjusted model excluding CD4			Model B—adjusted model including CD4		
		***n***	***N***	Median (IQR)/%	***n***	***N***	Median (IQR)/%	OR	95% CI	***p***	AOR	95% CI	***p***	AOR	95% CI	***p***
**Demographics**																
Age, years (for every 10-year increase)			2755	34 (29–41)		83	39 (31–49)	1.44	(1.19–1.73)	< 0.001						
Sex and pregnancy status	Pregnant	517	520	99%	3	520	1%	1.00	–	–	1.00	–	–	1.00	–	–
	Female non-pregnant	1379	1418	97%	39	1418	3%	4.87	(1.66–14.3)	0.004	2.45	(0.76–7.88)	0.133	2.04	(0.68–6.09)	0.201
	Male	859	900	95%	41	900	5%	8.23	(2.72–24.91)	< 0.001	5.47	(1.49–20.17)	0.011	4.35	(1.27–14.88)	0.019
Marital status	Married/civil union	292	300	97%	8	300	3%	1.00	–	–						
	Single	2367	2441	97%	74	2441	3%	1.14	(0.65–2)	0.646						
	Widowed/divorced	96	97	99%	1	97	1%	0.38	(0.05–3.14)	0.369						
Smoking history	Never	2262	2321	97%	59	2321	3%	1.00	–	–						
	Current/ex-smoker	493	517	95%	24	517	5%	1.87	(1.11–3.15)	0.019						
Employed	Employed	1233	1270	97%	37	1270	3%	1.00	–	–						
	Unemployed	1522	1568	97%	46	1568	3%	1.01	(0.68–1.48)	0.971						
Education	None	188	196	96%	8	196	4%	1.00	–	–						
	Primary	664	687	97%	23	687	3%	0.81	(0.36–1.85)	0.610						
	Secondary	1687	1734	97%	47	1734	3%	0.65	(0.31–1.39)	0.265						
	Higher	216	221	98%	5	221	2%	0.54	(0.23–1.26)	0.300						
**HIV/TB history**																
Previous TB treatment	No	2489	2561	97%	72	2561	3%	1.00	–	–						
	Yes	266	277	96%	11	277	4%	1.43	(0.74–2.77)	0.290						
Number of WHO TB symptoms	0	1955	1975	99%	20	1975	1%	1.00	–	–	1.00	–	–	1.00	–	–
	1	407	427	95%	20	427	5%	4.80	(2.8–8.23)	< 0.001	3.39	(1.88–6.09)	< 0.001	3.16	(1.84–5.43)	< 0.001
	2	188	202	93%	14	202	7%	7.28	(3.58–14.8)	< 0.001	4.03	(1.82–8.92)	0.001	3.64	(1.63–8.12)	0.002
	3 or 4	205	234	88%	29	234	12%	13.83	(9.04–21.13)	< 0.001	5.05	(3.31–7.7)	< 0.001	4.68	(2.93–7.48)	< 0.001
**Clinical characteristics**																
WHO stage	I/II	2441	2484	98%	43	2484	2%	1.00	–	–	1.00	–	–	1.00	–	–
	III/IV	314	354	89%	40	354	11%	7.23	(3.87–13.52)	< 0.001	2.57	(1.32–4.99)	0.005	2.47	(1.24–4.89)	0.010
CD4 (per 10-cell increase)^a^			2755	249 (149–317)		83	98 (41–218)	0.94	(0.9–0.98)	0.002				0.98	(0.95–1.01)	0.211
Weight (per 1-kg increase)^b^			2755	59 (52–68)		83	51 (45–60)	0.96	(0.93–0.98)	0.001						
BMI (per 1-unit increase)^b^			2755	21.6 (19.0–25.0)		83	19.0 (17.0–21.8)	0.86	(0.79–0.94)	0.001						
Hemoglobin (per 1-g/dL increase)			2755	11.9 (10.5–13.3)		83	9.9 (8.5–11.7)	0.69	(0.61–0.79)	< 0.001	0.73	(0.65–0.81)	< 0.001	0.74	(0.67–0.81)	< 0.001
Temperature (per 1 °C increase)^c^			2755	36.2 (35.8–36.5)		83	36.5 (36.0–37.0)	2.09	(1.47–2.96)	< 0.001	1.26	(0.96–1.65)	0.092	1.25	(0.94–1.67)	0.127
Heart rate (per 1 beat/min increase)^d^			2755	84 (75–94)		83	102 (85–121)	1.05	(1.04–1.06)	< 0.001						
Respiratory rate (per 1 breath/min increase)			2755	18 (18–20)		83	20 (18–22)	1.02	(1.01–1.04)	0.009						

*Abbreviations*: *ART* antiretroviral therapy, *TF* transfer-out, *CI* confidence interval, *WHO* World Health Organization, *BMI* body mass index, *OR* odds ratio, *AOR* adjusted odds ratio, *IQR* interquartile range

^a^Due to non-linearity in the association between CD4 and log odds of death, CD4 was modeled as two terms (term 1 = *X* − .2432641563 and term 2 = X*ln(*X*) + .3438800025, where *X* = CD4/1000). Output shown is for the linear term. The *p* value associated with each CD4 term was < 0.001

^b^BMI and weight were correlated, and BMI was retained as the preferred candidate variable. However, BMI was eliminated from the backward stepwise regression at *p* > 0.01

^c^Due to non-linearity in the association between temperature and log odds of death, temperature was modeled as two terms (term 1 = temperature^3 − 47,148.67774 and term 2 = temperature^3*ln (temperature) − 169,123.2696). Output shown is for the linear term. The *p* value associated with each squared term for temperature was < 0.001

^d^Heart rate was excluded as a candidate variable due to correlation with temperature

The final multivariable model A (which simulated the situation where CD4 is unavailable) included sex, number of WHO TB symptoms, WHO disease stage, hemoglobin concentration (continuous, linear term), and temperature (modeled as two transformed terms following output from the multivariable fractional polynomial analysis) (Table 2). In the final multivariable model B (which simulated the situation where CD4 is available), the same variables included in model A plus CD4 were included (Table 2).

### Internal validation of final regression models

**Fig. 2:**
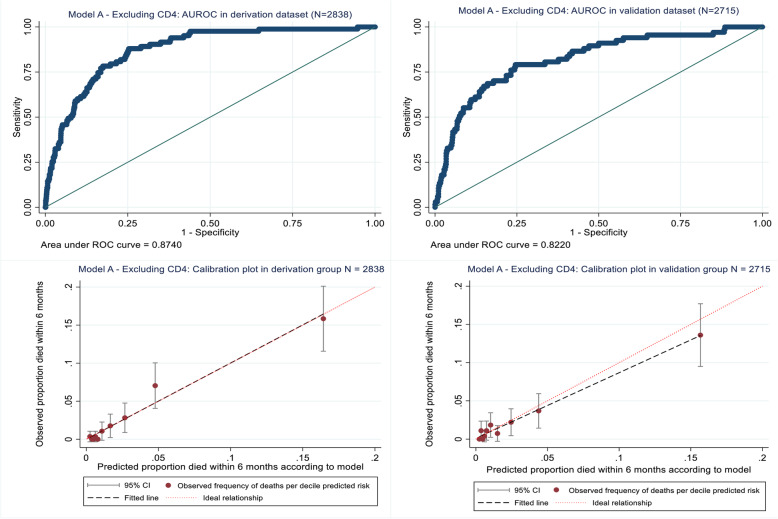
Model A (excluding CD4) development and performance in the internal derivation and validation datasets respectively

The Hosmer-Lemeshow statistics for model A (excluding CD4) on both the derivation (*p* = 0.381) and validation (*p* = 0.210) datasets indicated good model fit (see Additional file 4, table showing results of Hosmer-Lemeshow tests). Similarly, the calibration curves (Fig. 2) indicate adequate prediction performance for the 10 risk groups in terms of the predicted number of deaths within 6 months of ART versus the observed number of deaths. In addition, the AUROC curve values for the derivation (0.874) and validation (0.822) datasets indicated excellent discrimination (Fig. 2).

**Fig. 3:**
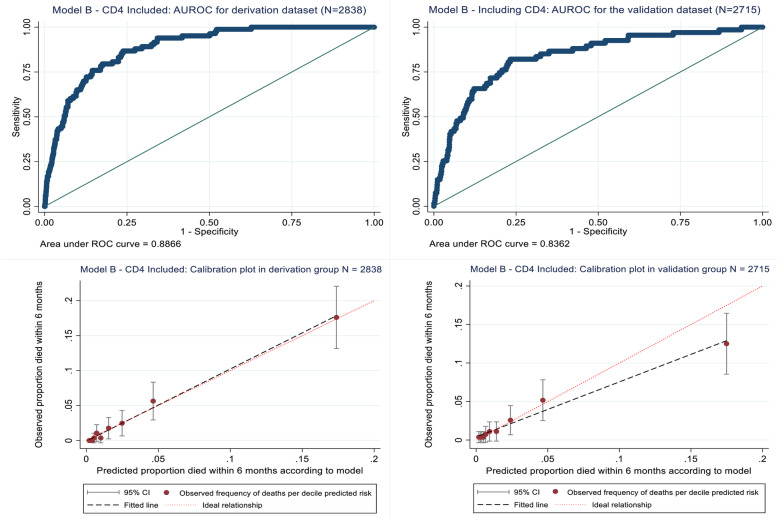
Model B (including CD4) development and performance in the internal derivation and validation datasets respectively

The Hosmer-Lemeshow statistics for model B (including CD4) on both the derivation (*p* = 0.735) and validation (*p* = 0.677) datasets also indicated good model fit (see Additional file 4, table showing results of Hosmer-Lemeshow tests), with calibration curves (Fig. 3) indicating adequate prediction performance for the 10 risk groups. However, in the highest risk group (risk group 10), model B overestimated mortality risk in the validation dataset, with 48 deaths predicted but only 34 observed (see Additional file 4, table showing results of Hosmer-Lemeshow tests). In addition, the AUROC curve values for the derivation (0.887) and validation datasets (0.836) indicated excellent discrimination (Fig. 3).

### Transformation from the regression model to clinical score

We used WHO advanced disease classifications for WHO stage (stage III or IV), and CD4 count (< 200 cells/μL). Anemia severity in adults was classified according to WHO criteria as follows [25]: no anemia was defined as hemoglobin ≥ 13.0 g/dL for men, ≥ 12.0 g/dL for non-pregnant females, and ≥ 11.0 g/dL for pregnant females; mild/moderate anemia was defined as 8.0 to < 13.0 g/dL for men, 8.0 to < 12.0 g/dL for non-pregnant females, and 7.0 to < 11.0 g/dL for pregnant females; and severe anemia was defined as < 8.0 g/dL for males and non-pregnant females and < 7.0 g/dL for pregnant females. Temperature was classified as ≤ 37.5 °C versus > 37.5 °C based on the observed distribution of mortality risk as measured temperature increased (see Additional file 5, figure of association between temperature and risk of death), and a common definition of a low-grade fever or higher (> 37.5 °C) [26]. The multivariable model with categorization of these continuous variables in the derivation dataset is presented in Table 3. Because heart rate might be more easily available in LMIC settings than temperature, we also created an alternate clinical score where heart rate replaced temperature (see Additional file 6, table showing alternate clinical score). In this alternate score, we used heart rate cut-offs of > 120 versus ≤ 120 beats/minute, which were informed by both prior published literature [22, 27] and observed inflection points in the association between heart rate and risk of death (see Additional file 5, figure of association between heart rate and risk of death).

**Table 3:** Multivariable model and clinical score generation from the derivation dataset (*N* = 2838)

		Predictor—model A (excluding CD4)					Predictor—model B (including CD4)				
		AOR	95% CI	***p*** value	***ß*** coefficient	Score	AOR	95% CI	***p*** value	***ß*** coefficient	Score
Sex and pregnancy status	Female (pregnant)	1.00	–	–	–	0	1.00	–	–	–	0
	Female (non-pregnant)	1.94	(0.58–6.50)	0.283	0.66	1	1.71	(0.53–5.52)	0.373	0.53	1
	Male	3.54	(0.95–13.26)	0.060	1.26	2	2.93	(0.82–10.44)	0.097	1.08	2
Number of WHO TB symptoms	0	1.00	–	–	–	0	1.00	–	–	–	0
	≥ 1	3.65	(2.24–5.97)	< 0.001	1.30	2	3.33	(2.06–5.38)	< 0.001	1.20	2
WHO stage	I/II	1.00	–	–	–	0	1.00	–	–	–	0
	III/IV	2.72	(1.42–5.20)	0.003	1.00	2	2.55	(1.32–4.92)	0.005	0.94	2
Temperature at enrollment	≤ 37.5 °C	1.00	–	–	–	0	1.00	–	–	–	0
	> 37.5 °C	3.39	(1.65–6.96)	0.001	1.22	2	3.37	(1.56–7.26)	0.002	1.21	2
CD4 count	≥ 200/μL	–	–	–	–	N/A	1.00	–	–	–	0
	< 200/μL	–	–	–	–	N/A	2.05	(1.20–3.50)	0.009	0.72	1
Anemia status^a^	No anemia	1.00	–	–	–	0	1.00	–	–	–	0
	Mild/moderate anemia	5.03	(2.57–9.87)	< 0.001	1.62	2	4.58	(2.37–8.84)	< 0.001	1.52	3
	Severe anemia	9.42	(3.43–25.89)	< 0.001	2.24	3	8.02	(3.04–21.14)	< 0.001	2.08	4

*Abbreviations*: *AOR* adjusted odds ratio, *CI* confidence interval, *WHO* World Health Organization

^a^Anemia severity was classified according to World Health Organization criteria as follows: no anemia, hemoglobin level of ≥ 13.0 g/dL for men, ≥ 12.0 g/dL for non-pregnant females, and ≥ 11.0 g/dL for pregnant females; mild/moderate anemia, 8.0 to < 13.0 g/dL for men, 8.0 to < 12.0 g/dL for non-pregnant women, and 7.0 to < 11.0 g/dL for pregnant women; and severe anemia, < 8.0 g/dL for males and non-pregnant females and < 7.0 g/dL for pregnant women

**Fig. 4:**
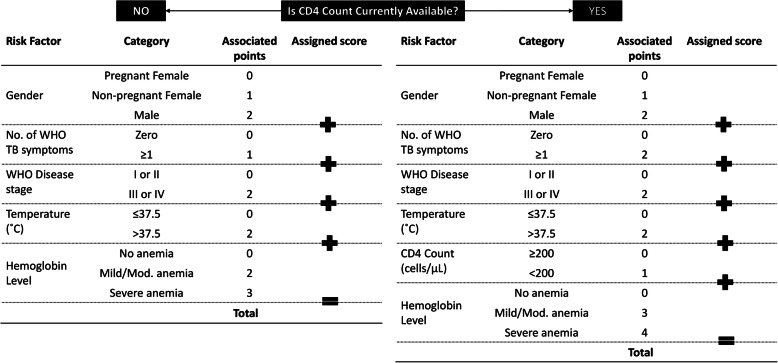
CD4-independent and CD4-dependent clinical score cards

Model A, categorized in this way, retained statistically excellent discrimination in both derivation (AUROC 0.867) and validation datasets (AUROC 0.818), and the Hosmer-Lemeshow statistic *p* values were 0.269 in the derivation and 0.334 in the validation datasets indicating good calibration. Similarly, model B AUROC statistics were 0.874 in the derivation and 0.830 in the validation datasets, with Hosmer-Lemeshow statistic *p* values of 0.367 and 0.307 in the derivation and validation datasets, respectively, indicating good model fit. The clinical scores that could be used in clinic settings to identify those at risk of early 6-month mortality, depending on the availability of CD4 count, are illustrated in Fig. 4.

### External validation of risk scores

**Fig. 5:**
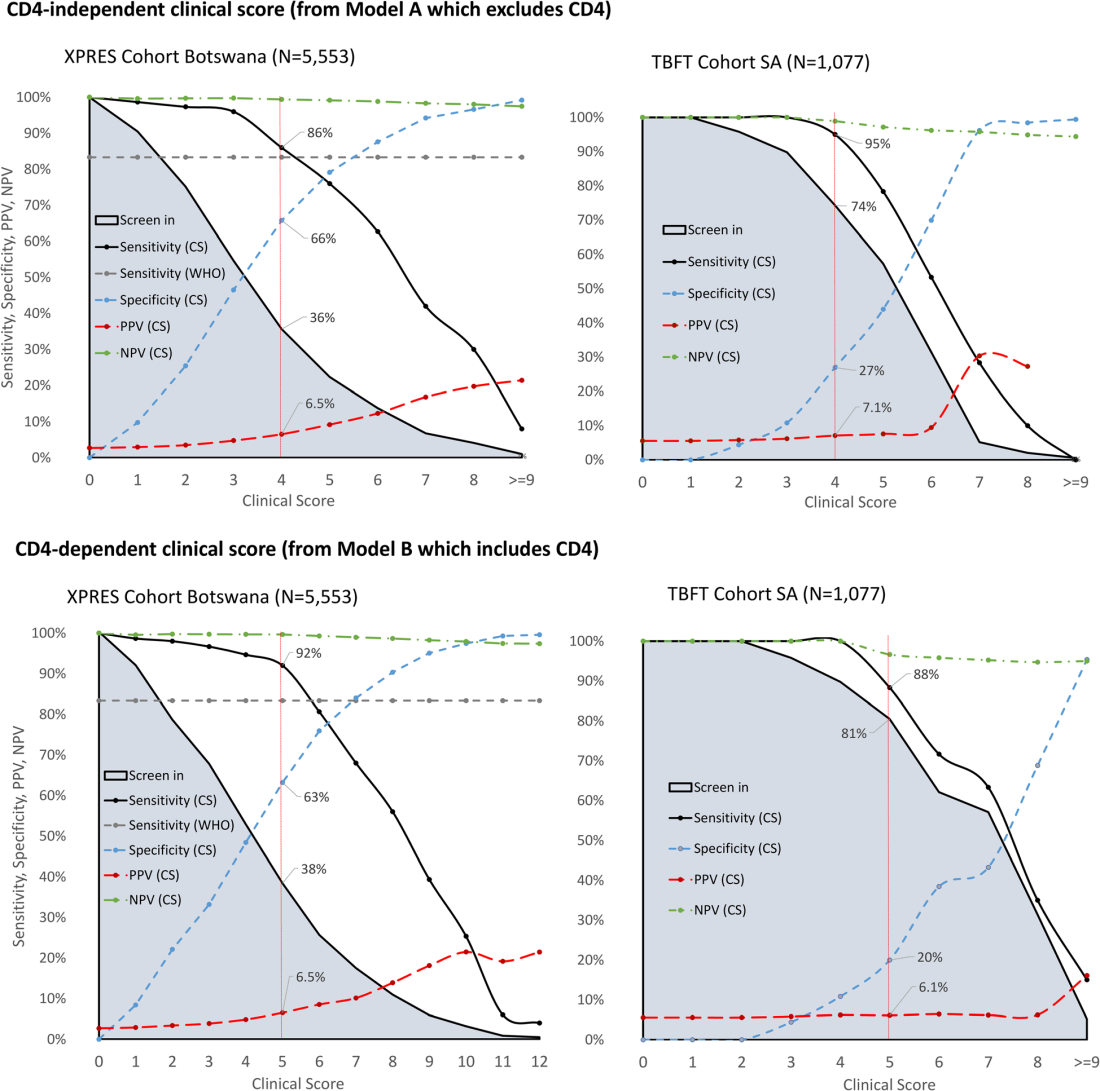
Sensitivity, specificity, PPV, and NPV of clinical score in predicting 6-month mortality in XPRES dataset (*N* = 5553) and external validation TB Fast Track Dataset (*N* = 1077) for models A (excluding CD4) and B (including CD4)

The clinical score for each predictor was generated, and the possible range for the total score was 0 to 11 for model A and 0 to 13 for model B (see Additional file 7, tables showing performance of clinical scores). Figure 5 shows the performance of the two clinical scores at different cut-offs, in terms of sensitivity, specificity, negative predictive value (NPV), positive predictive value (PPV), and percentage of enrollees screened into ART care intensification.

For the CD4-independent clinical score derived from model A, (Fig. 5) among XPRES enrollees, a clinical score of ≥ 4 would screen in 36% of ART enrollees into a care intensification pathway, providing 86% sensitivity and 66% specificity in detecting those at risk for early mortality, whereas the WHO advanced disease eligibility criteria (CD4 < 200/μL or WHO stage III/IV) would screen in 44% of ART enrollees, providing 83% sensitivity and 58% specificity. Notably, if the WHO advanced disease eligibility criterion of WHO stage III/IV only was used since CD4 is unavailable, 12% of ART enrollees would be screened into an ART care intensification pathway, with only 48% sensitivity in detecting 6-month mortality and 89% specificity. All 72 XPRES patients with WHO stage III/IV who died by 6 months of ART would also be screened into intensification of care pathways using a clinical score cut-off of ≥ 4. Among TBFT enrollees, the clinical score of ≥ 4 would screen in 74% of ART enrollees, providing 95% sensitivity and 27% specificity in detecting early mortality, versus the WHO advanced disease eligibility criteria which would screen in 100% of ART enrollees, with 100% sensitivity but 0% specificity.

For the CD4-dependent clinical score derived from model B, a clinical score of ≥ 5 would screen in 38% of ART enrollees into a care intensification pathway, providing 92% sensitivity and 63% specificity in detecting those at risk for early mortality. Ninety-seven percent (121 of 125) XPRES patients with either a CD4 < 200/μL or WHO stage III/IV who died by 6 months would be screened into intensification of care pathways using a clinical score ≥ 5. Among TBFT enrollees, the clinical score of ≥ 5 would screen in 81% of ART enrollees, providing 88% sensitivity and 20% specificity in detecting early mortality. For both CD4-dependent and CD4-independent clinical scores, screening accuracy characteristics were very similar when heart rate replaced temperature in the clinical score (see Additional file 8, figure of screening accuracy for the alternate clinical scores).

The AUROC for CD4-independent (0.845) and CD4-dependent (0.852) clinical scores remained high for XPRES enrollees but was low for TB FT enrollees (0.568 for CD4-independent and 0.569 for CD4-dependent scores) (see Additional file 9, figure of AUROC for clinical score performance).

**Fig. 6:**
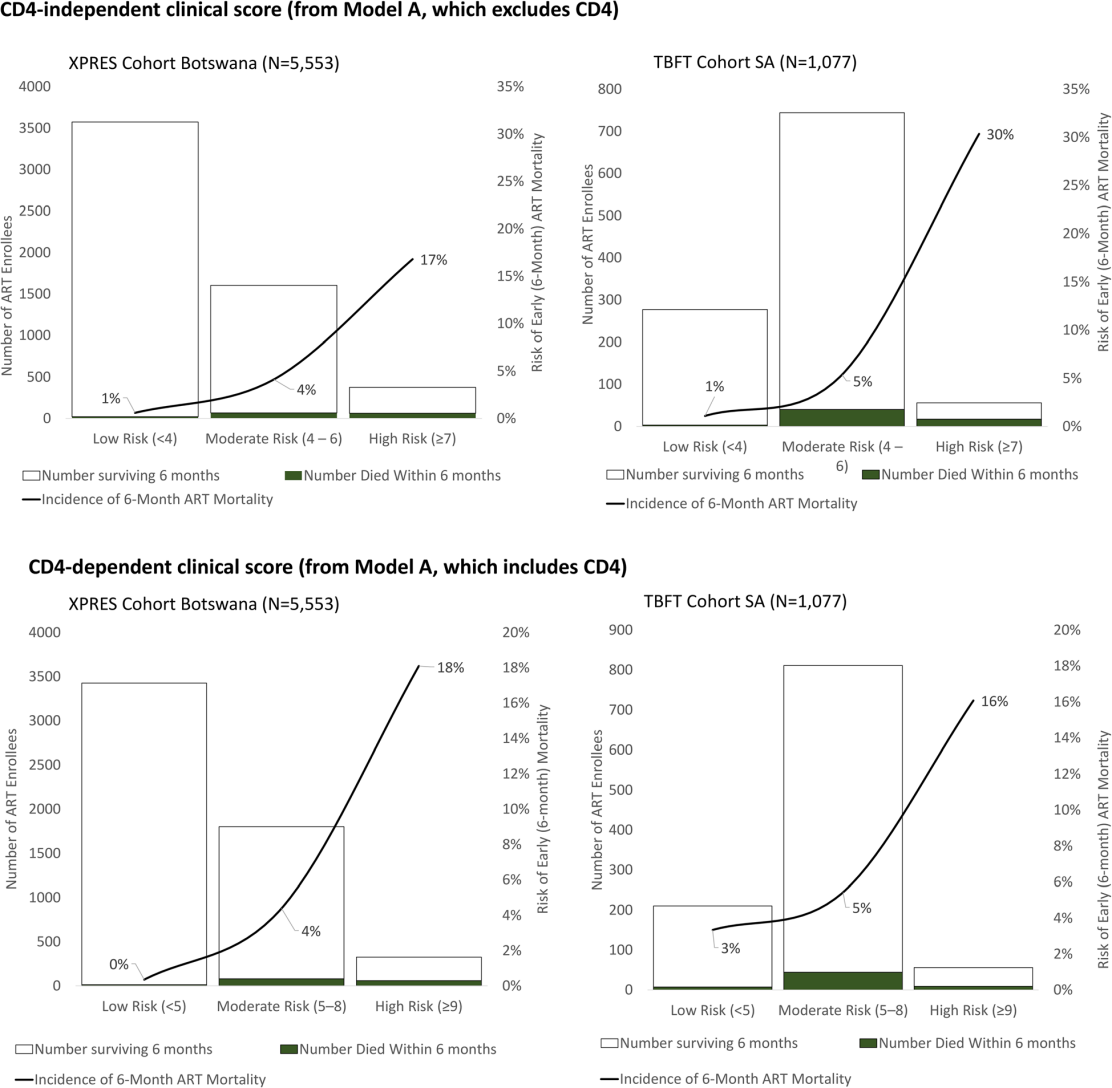
Distribution of risk scores and 6-month mortality risk in the XPRES dataset (*N* = 5553) and external validation TB Fast Track Dataset (*N* = 1077) for models A (excluding CD4) and B (including CD4)

**Fig. 7:**
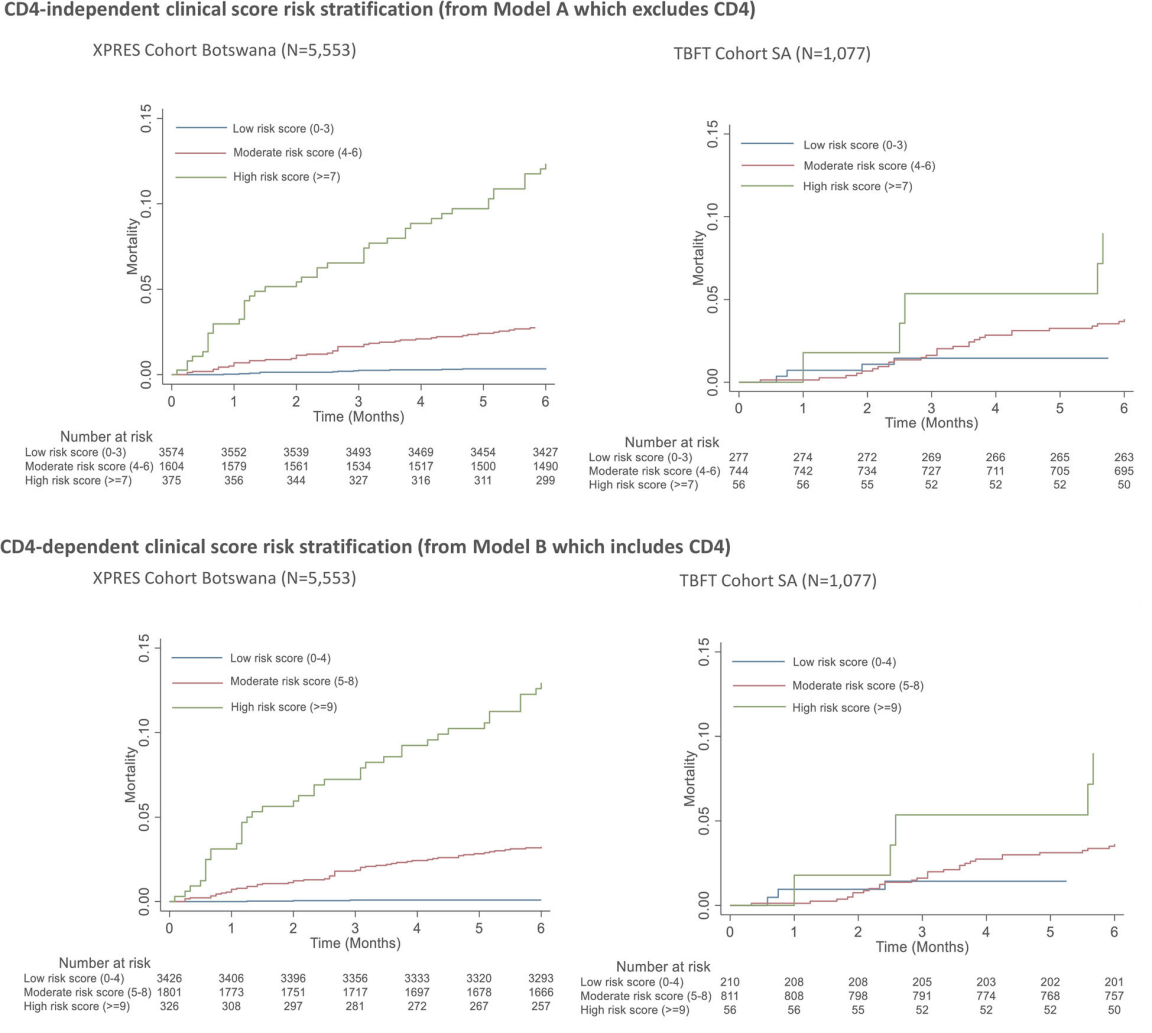
Survival curves stratified by risk scores in the XPRES dataset (*N* = 5553) and external validation TB Fast Track Dataset (*N* = 1077) for models A (excluding CD4) and B (including CD4)

For the CD4-independent clinical score, risk scores were grouped into low- (< 4), moderate- (4–6), and high-risk categories (≥ 7) (Fig. 6), with 6-month low-, moderate-, and high-risk group incidence percentages being 1%, 4%, and 17% among XPRES enrollees and 1%, 5%, and 30% among TBFT enrollees. Similarly, for the CD4-dependent clinical score, risk scores were grouped into low- (< 5), moderate- (5–8), and high-risk categories (≥ 9) (Fig. 6), with 6-month low-, moderate-, and high-risk group mortality percentages being 0%, 4%, and 18% for XPRES enrollees and 3%, 5%, and 16% for TBFT enrollees. Figure 7 shows K-M failure curves of mortality over the first 6 months of ART according to the low-, moderate-, and high-risk groups, indicating that specific populations of moderately high- and high-risk groups, in high need of care intensification, were differentiated by the respective clinical scores.

## Discussion

To our knowledge, these are the first externally validated clinical scores for ART care intensification generated for SSA. The scores, which have superior screening accuracy characteristics in predicting early mortality risk than WHO-recommended advanced disease eligibility criteria, are not dependent on CD4 testing access, can differentiate mortality risk into three risk groups, could improve access to evidence-based early ART care packages, improve efficiency of advanced disease DSD models, and facilitate improved differentiated care [9].

The CD4-independent clinical score, designed for settings where CD4 is unavailable at ART intitiation, with a cut-off score of ≥ 4 was largely as sensitive (86–95%) in screening in persons at risk of death by 6 months as the current WHO advanced disease eligibility criteria (83–100%) and nearly twice as sensitive as WHO eligibility criteria that would rely on WHO stage alone (48%). Compared with the CD4-based WHO advanced disease eligibility criteria, the CD4-independent clinical score had higher specificity and would screen 8–26% fewer ART enrollees into intensified care pathways, suggesting the screening tool could also increase efficiency of investments in DSD models for advanced disease. Therefore, in the many settings in SSA that lack access to rapid CD4 testing, the CD4-independent clinical score should be considered for scale-up to facilitate early ART care intensification, with the potential for reductions in early ART mortality [11]. In addition, in those settings where CD4 is available, using the CD4-dependent clinical score with a cut-off score of ≥ 5 could have similar or increased sensitivity and superior specificity compared with WHO advanced disease eligibility criteria, with the potential to both reduce early ART mortality and improve efficiency of DSD algorithms.

In contrast to current WHO guidelines, which recommend only the use of CD4 count and WHO HIV disease staging to identify patients at high risk for morbidity and mortality, our composite risk score provides both more comprehensive and specific information on the magnitude of risk for each patient by integrating additional objective variables into the assessment [9]. The additional variables included in our score are both clinical and demographic. The clinical variables of WHO TB symptom screen, temperature, and anemia severity are known to be associated with serious comorbidities that significantly increase early mortality risk, while the demographic variable in the scores (the gender variable of male, female non-pregnant, and female pregnant) captures important generalizable differences in early mortality risk in SSA, which are due to both psychosocial and biological factors [28, 29]. Our risk scores are careful to be simple (5 or 6 variables assessed), use objective covariates rather than variables that are more open to interpretation, and use variables that should be available, or could easily be made available, at the POC in LMIC. Our score could be relatively easily included in paper medical records relevant for the first HIV clinic or ART initiation visit and should not require a calculator, unlike clinical scores developed for resource-rich settings that require either electronic medical record or website access to calculate the score (e.g., the Veterans Aging Cohort Study (VACS) or EuroSIDA scores) [30–32].

The hemoglobin concentration variable in our scores is more available in LMIC than POC CD4 testing, although scale-up of CD4 testing is needed and ongoing. Notably, WHO has long designated hemoglobin testing one of four essential laboratory services in SSA [33], and hemoglobin tests are the most commonly performed laboratory test globally [34, 35]. For example, in Malawi, one of the most resource-constrained countries in the world which is ranked 172 out of 189 countries on the human development index, hemoglobin testing through point-of-care HemoCue® [34] or the WHO Hemoglobin Color Scale is relatively widely available [35]. In the 2014 Malawi national health facility survey to assess access to diagnostic tests, 82% of hospitals had access to hemoglobin testing, with access only superseded by malaria diagnostic testing (95%) and HIV diagnostic testing (95%), whereas CD4 testing was only available in 43% of hospitals [36]. Across all health facility types (hospitals, health centers, clinics, and health posts), hemoglobin testing was three times more widely available than CD4 testing [36], although there is a need for scale-up of both tests. In addition to being currently more widely accessible in SSA than CD4 testing, POC hemoglobin testing is currently easier to scale up than CD4 testing. Available POC hemoglobin measurement devices tend to be durable, easy to use, and not reliant on electricity supply, and require minimal training and supervision [34] while also providing good accuracy in LMIC [37, 38]. To date, these POC hemoglobin devices have been less expensive than currently available POC CD4 systems and are useful for non-HIV-related care (e.g., <$100/POC hemoglobin measurement device and $0.12–0.75/test [34] vs. about $7430/POC CD4 device and about $8.70/test [39]). Both CD4 testing and hemoglobin testing are important at the point of care, and less expensive POC CD4 lateral flow assays and transcutaneous spectrophotometry solutions for hemoglobin level measurement may become available in the future [40–42].

Additional advantages of developing clinical scores with a variety of cut-offs are that it allows program managers to choose cut-offs with associated screening accuracy characteristics, allowing program managers to choose cut-offs based on funding availability, by trading sensitivity for improved specificity [9].

Another potential advantage of the combined clinical score over the WHO advanced disease criteria is the ability to differentiate three risk groups (low, moderate, and high), with the highest risk group having 6-month mortality rates of 16–30% versus 0–3% in the low and 4–5% in the moderate risk groups. While all patients with moderate or high scores might benefit from standardized outpatient intensified early ART care, patients in the highest risk group might be candidates for additional interventions to help navigate the relatively complex time of early ART. During this time, clinicians need to rapidly search for, diagnose, or rule out comorbidities, and both choose and time appropriate therapies, all within the context of ART-driven immune reconstitution [43, 44]. Our clinical score could be used to inform a clinical trial of such interventions.

Moderate to severe anemia was a stronger predictor than CD4 count and overall was the strongest predictor of early ART mortality in our cohort, similar to other studies in SSA [21, 45, 46]. Anemia is the most common hematological complication of HIV disease among PLHIV [47] and develops through several mechanisms including direct HIV infection of hematopoietic progenitor cells, dysregulated erythropoiesis through indirect effects of proinflammatory cytokines, and through anemia of chronic disorders (ACD), which is thought to be the most common pathway [48]. ACD is driven by hepatic expression of hepcidin, an acute phase reactant that causes iron to be diverted from the circulation and sequestered within cells of the reticuloendothelial system through downregulation of ferroportin channels [49]. TB also drives ACD through this hepcidin-ferroportin interaction [49, 50]. In turn, sequestration of iron inside macrophages and T cells might support both intracellular mycobacterial growth [46, 49] and HIV viral replication [51], showing the potential for rapid worsening of HIV, TB, and severe hepcidin-driven anemia. Therefore, although ART is the most important treatment of HIV-associated anemia, early treatment of any associated co-infections is crucial [46]. In a separate analysis, we show that moderate to severe anemia was also predictive of active TB infection in the XPRES cohort, similar to other analyses [46]. Given the strong association between moderate to severe anemia, early mortality, and active TB, which is the most common cause of early mortality in SSA [52], the scores associated with observed moderate-severe anemia in this analysis (2–4 points) appropriately bring the total clinical score very close to the threshold for ART care intensification. Per current WHO guidelines, care intensification should include further investigations for TB, especially disseminated TB, through the use of the urine TB-LAM assay and Xpert MTB/RIF [46, 50, 53, 54].

Another notable finding is that measured temperature at > 37.5 °C at ART initiation was strongly predictive of early ART mortality, independent of the WHO TB symptom screen for fever or night sweats, which was also predictive of mortality. This indicates the importance of objective measures of fever in addition to patient history [23]. In addition, our analysis shows that in those settings where measured temperature measurement is not available, measured heart rate (> 120 versus ≤ 120/min) is a suitable replacement variable. Notably, some of the key inflammatory cytokines that drive hepcidin release and fever are the same (e.g., interleukin (IL)-6, tumor necrosis factor which stimulates IL-6 release, interferons, and microbial-derived Toll-like receptors) and are important for both pathways [49, 55]. Disseminated undiagnosed TB or TB diagnosed late is the most common infectious cause of death among PLHIV in sub-Saharan Africa, accounting for about 40% of deaths [52]. However, a recent autopsy study of causes of death among new HIV clinic enrollees in SA found that 59% of decedents had evidence of two or more concurrent infections [56]. Most bacterial infections were due to common pathogens, such as *Klebsiella* spp., *Salmonella* spp., *Haemophilus influenzae*, and *Staphylococcus aureus*, while cryptococcal infection was found in 13% [56]. Targeting an antimicrobial package of interventions to patients who screen positive for our proposed clinical scores, such as the package of interventions recommended by WHO or trialed in the REALITY trial (continuous trimethoprim–sulfamethoxazole, ≥ 12 weeks of isoniazid–pyridoxine (once active TB is ruled out), 12 weeks of fluconazole, 5 days of azithromycin, and a single dose of albendazole), could significantly reduce mortality for patients who screen positive [11].

The prognostic importance of male gender in predicting mortality was correlated with older age and smoking history in our model, and we chose to include the single gender variable rather than two additional variables (age ≥ 55 and smoking) in the CD4-dependent clinical score to make the most parsimonious clinical score and because male gender is a more generalizable predictor of poor outcomes in SSA [29, 57, 58]. In addition, similar to many ART programs in SSA [20, 59], pregnant women in XPRES, who were (1) more likely to be diagnosed at an earlier disease stage through routine testing at antenatal care and (2) able to initiate ART immediately once diagnosed unlike non-pregnant women diagnosed with HIV at the time [15], had lower mortality than non-pregnant women starting ART in bivariate analysis [20, 59]. However, if ART programs in SSA are able in the future to achieve earlier testing and ART initiation for male and non-pregnant female PLHIV, it is likely gender and pregnancy status could become less important predictors, while predictors like smoking and older age will become more important [57]. Although smoking is not part of the clinical score, this article provides additional evidence for the need for tobacco smoking reduction programs for PLHIV, separate or included in early ART care intensification algorithms, to minimize not only the risk of ischemic cardiovascular diseases but also the risk of malignancies and bacterial infections, including TB [60].

Strengths of this study include the use of data from prospective cohorts nested within clinical trials, meaning there was minimal missing covariate data and strong ascertainment of the primary outcome of interest (6-month ART mortality) (e.g., only one patient was LTFU from the XPRES cohort and was excluded from this analysis [16]). Additional strengths include the relatively high screening accuracy in both the XPRES and TBFT cohorts, from two geographically separate cohorts, with very different cohort characteristics (e.g., XPRES enrollees represent general outpatient ART enrollees while TBFT enrollees had homogenously low CD4 counts (< 150/μL)). Notably, discrimination, as measured by the AUROC of the clinical scores, was lower in the TBFT than in the XPRES cohort, but at the chosen clinical score cut-offs, the clinical score still provided similar sensitivity and superior specificity in predicting early ART mortality compared with the WHO advanced disease eligibility criteria. The lower discriminatory capacity of the clinical scores in the TBFT cohort is not surprising given the TBFT cohort reflects a relatively homogenous ART population with advanced HIV disease. Notably, while in the XPRES cohort 6% of ART enrollees were newly diagnosed and treated for TB, in the TBFT cohort 62% were treated for TB through a risk-based TB-treatment algorithm [14], suggesting that the risk score is likely to be generalizable across a wide range of new ART enrollee cohorts. However, additional validation exercises are needed and planned to further assess generalizability.

Limitations include that the risk score has not yet been validated in a cohort enrolled under HIV test-and-treat guidelines, something which is planned in the near future. Other limitations include the fact that while the gender and pregnancy variable is relevant in SSA and many resource-limited settings, it is not generalizable to cohorts in resource-rich settings like the USA and Europe, where males often have better outcomes than female ART enrollees. Although the specificity of the clinical scores is superior to the WHO advanced disease eligibility criteria, a substantial percentage of ART enrollees (36–38% in the XPRES cohort) would be screened into receiving an advanced disease care package, which would require a monitoring system to assess implementation fidelity. In addition, these screening tools were validated in clinical trial cohorts that received relatively intensive TB screening and treatment services, and therefore, those that died did so despite access these services. Finally, although the clinical score is highly sensitive in screening in almost all patients with low CD4 count and advanced WHO disease stage at risk of death into intensification of care pathways, and has superior or similar sensitivity to current WHO advanced disease screening criteria, it is possible for some patients with a very low CD4 count and advanced WHO stage to have a clinical score that falls below the specified cut-off, and clinical discretion to screen these rare patients missed by the screening tool into intensification of care pathways is warranted.

## Conclusions

In conclusion, where CD4 testing is not available in similar LMIC, especially in SSA, the CD4-independent risk score should be strongly considered for scale-up to facilitate early ART care intensification, with the potential for significant reductions in early ART mortality if targetted individuals are provided with evidence-based care packages [11]. For clinics where CD4 count is available, the use of the CD4-dependent clinical score could improve both sensitivity and specificity over WHO advanced disease eligibility criteria, with the potential to reduce early ART mortality and improve efficiency of DSD algorithms. Finally, further research to understand best management of ART enrollees enrolled in the highest risk categories is warranted to further explore mortality reduction interventions. Together, these actions could help drive progress to AIDS 2030 goals of zero AIDS deaths in the region of the world with the highest HIV/AIDS-associated mortality.

## Supplementary information

**Additional file 1.** Table showing HIV care clinical follow-up of clients in the Botswana XPRES cohort (2010–2015).

**Additional file 2.** Text showing XPRES enrollment and follow-up procedures.

**Additional file 3.** Tripod checklist for prediction model development and validation.

**Additional file 4.** Table showing Hosmer-Lemeshow tests for calibration of final models A (CD4 excluded) and B (CD4 included).

**Additional file 5.** Figure showing association between 6-month ART mortality risk and (a) temperature and (b) heart rate, at ART initiation.

**Additional file 6 **Table showing multivariable model and clinical score generation from the derivation dataset with heart rate replacing the measured temperature variable (*N* = 2838).

**Additional file 7.** Tables showing performance of clinical score in derivation and validation datasets for Models A (excluding CD4) and B (including CD4).

**Additional file 8.** Figure showing alternate clinical score screening accuracy with the temperature variable replaced by heart rate.

**Additional file 9.** Figure showing area under the receiver operating characteristic curve for clinical score performance in combined XPRES dataset (N = 5553) and external validation TB Fast Track Dataset (N = 1077) for Models A (excluding CD4) and B (including CD4).

## Data Availability

The datasets generated and/or analyzed during the current study are not publicly available due to an IRB decision which was made in the interest of ensuring patient confidentiality but are available from the corresponding author on reasonable request. Per IRB guidance, the datasets will be anonymized before sharing.
