# Supplementary material for: Risk scores for predicting early antiretroviral therapy mortality in sub-Saharan Africa to inform who needs intensification of care: a derivation and external validation cohort study
Source: BMC Med. 2020 Nov 9;18:311. doi: 10.1186/s12916-020-01775-8 (PMC7650165; doi:10.1186/s12916-020-01775-8)
Supplement: Supplementary file 1 — Additional file 1. Table showing HIV care clinical follow-up of clients in the Botswana XPRES cohort (2010–2015). [file 12916_2020_1775_MOESM1_ESM.pdf]

**Additional file 1 - Table: HIV care clinical follow-up of clients in the Botswana XPRES cohort (2010-2015)**

| | | |
|---|---|---|
| **Pre-ART, CD4 >350** | **3 monthly** | Weight, CD4, TB screen |
| **ART** | **ART start** | Weight, CD4, TB screen, ALT/AST if NVP-based regimen, Hb if AZT-based regimen, Hepatitis B screen, creatinine if TDF-based regimen |
| | **2 weeks** | Weight, TB screen, ALT/AST if NVP-based regimen, Hb if AZT-based regimen |
| | **1 month** | Weight, TB screen, ALT/AST if NVP-based regimen, Hb if AZT-based regimen |
| | **3 months** | Weight, TB screen, ALT/AST if NVP-based regimen, Hb if AZT-based regimen, Viral load, creatinine if TDF-based regimen |
| | **6months** | Weight, TB screen, ALT/AST[a] if NVP-based regimen, Hb if AZT-based regimen, Viral load, CD4 |
| | **Quarterly[b]** | Weight, TB screen, Viral load and CD4 6 monthly, creatinine if TDF-based regimen 6 monthly |

Abbreviations: CD4, CD4 cell count; TB, tuberculosis; ALT, alanine transaminase; AST, aspartate aminotransferase; NVP, nevirapine; AZT, zidovudine; TDF, tenofovir;

[a]Routine ALT/AST not required after 6 months but may be requested by the clinician depending on the clinical situation.

[b]For those patients started on PI-based regimens, baseline and 12-monthly glucose (random or fasting) and total cholesterol/triglycerides are recommended.
