# Supplementary material for: Risk scores for predicting early antiretroviral therapy mortality in sub-Saharan Africa to inform who needs intensification of care: a derivation and external validation cohort study
Source: BMC Med. 2020 Nov 9;18:311. doi: 10.1186/s12916-020-01775-8 (PMC7650165; doi:10.1186/s12916-020-01775-8)
Supplement: Supplementary file 2 — Additional file 2. Text showing XPRES enrollment and follow-up procedures. [file 12916_2020_1775_MOESM2_ESM.pdf]

Additional file 2: XPRES Enrollment and Follow-up Procedures

Enrollment

At prospective cohort enrolment, research staff administered a standardized questionnaire, which captured demographic characteristics including age, sex, and pregnancy status, as well as clinical characteristics, including the WHO TB symptom screening rule for any current cough, fever, night sweats or weight loss, WHO HIV disease stage, height and weight, temperature in degrees centigrade, the most recent hemoglobin level, and the most recent CD4 count.

Interventions to strengthen ICF and active tracing

The ICF and active tracing interventions were strengthened through four key mechanisms: (1) additional human resources (study nurses) to support implementation, (2) additional training for clinic and laboratory personnel, (3) use of checklists and job aids to standardize implementation, and (4) regular supervisory visits to track adherence to ICF and tracing checklists.

Strengthened ICF

The above four interventions helped strengthen implementation of the following ICF cascade:

- Screening: Implementation of the WHO 4-symptom TB screening rule (i.e., screening for cough of any duration, fever, loss of weight, and night sweats) was strengthened for all prospective XPRES cohort enrollees.
- Screening positive: Clients were considered symptomatic if they screened positive for one or more of the four TB symptoms.
- Sputum sample collection: For patients screening TB symptom positive, at least two same-day, on-the-spot (spot) sputum samples were recommended for collection. In addition, if feasible for the patient, a morning sputum the day after screening positive for TB symptoms was recommended

along with a third spot sputum upon arrival at the clinic. As part of strengthened ICF, a previously published job-aid was used by study nurses to inform the patient how to collect quality sputum samples.

- Sputum sample testing:
  - Sputum smear microscopy: At study initiation, sputum smear microscopy was still the standard first-line TB diagnostic test for PLHIV screening TB symptom positive. Therefore, laboratory personnel at the 13 laboratories serving the 22 study clinics received refresher training on Ziehl-Neelsen staining for sputum-smear microscopy.
  - Xpert MTB/RIF (Xpert) rollout: During the XPRES trial, Xpert was rolled out in a phased manner, replacing sputum smear microscopy as the initial TB diagnostic test for PLHIV screening TB symptom positive. Prior to activation of the Xpert device, all laboratory personnel were trained for Xpert implementation.
  - Additional tests: When all four sputum samples were available, the morning sputum sample and second spot sputum sample for all symptomatic clients were sent to the national TB reference laboratory (NTRL) for culture using mycobacteria growth indicator tubes (MGIT). Turnaround times for sputum culture were expected to be >49 days per standard of care at the time.
- Result return to the clinicians and patients: In all phases, sputum test results were returned to the clinics, with clinicians responsible for informing the patients. In the XPRES prospective phases included in this study, study nurses were trained to work with laboratories to ensure the turnaround time from sample collection to result return to the clinic was ≤4 days for sputum-smear microscopy and ≤2 days for Xpert testing. In the EC and EC+X phases, nurses were trained to inform patients of positive TB diagnoses the same day via phone, or if unreachable by phone, by active tracing to the household.
- ICF cascade monitoring: Indicators monitoring implementation of the ICF cascade were collected and used to inform supervision visits.

Strengthened active tracing

As part of the active tracing intervention, for all patients ≥1 day late for a clinic appointment, study nurses conducted up to five telephone calls and two home visits in attempts to return these clients to care. XPRES participants were followed for 12 months, or until the end of TB treatment, whichever was later. The final follow-up visits for XPRES enrollees were in June 2015.
