# Supplementary material for: Risk scores for predicting early antiretroviral therapy mortality in sub-Saharan Africa to inform who needs intensification of care: a derivation and external validation cohort study
Source: BMC Med. 2020 Nov 9;18:311. doi: 10.1186/s12916-020-01775-8 (PMC7650165; doi:10.1186/s12916-020-01775-8)
Supplement: Supplementary file 4 — Additional file 4. Table showing Hosmer-Lemeshow tests for calibration of final models A (CD4 excluded) and B (CD4 included). [file 12916_2020_1775_MOESM4_ESM.pdf]

Additional file 4a: Hosmer-Lemeshow test for calibration of final model – Model A (CD4 excluded)

Derivation Dataset					Validation Dataset				
			Death					Death	
Decile	N	Cut off*	Observed**	Predicted†	Decile	N	Cut off*	Observed**	Predicted†
1	285	0.0034	1	0.7	1	272	0.0031	0	0.7
2	283	0.0045	0	1.1	2	271	0.0042	3	1
3	284	0.0057	0	1.4	3	273	0.0053	0	1.3
4	284	0.007	1	1.8	4	270	0.0066	1	1.6
5	283	0.0091	0	2.3	5	272	0.0085	3	2
6	284	0.0131	3	3.1	6	271	0.0122	5	2.8
7	284	0.021	5	4.7	7	272	0.0189	2	4.1
8	284	0.0345	8	7.6	8	271	0.031	6	6.7
9	284	0.0689	20	13.6	9	272	0.0619	10	11.9
10	283	0.7517	45	46.6	10	271	0.5941	37	42.6
Total	2838		83	83		2715		67	75

Derivation dataset Hosmer-Lemeshow chi2(8) = 8.56, p=0.3807
Validation dataset Hosmer-Lemeshow chi2(8) = 10.91, p=0.2069
* Upper boundary of predicted risk
**Observed = observed number dying within 6 months of ART initiation
†Predicted = expected number dying within 6 months of ART initiation

Additional file 4b: Hosmer-Lemeshow test for calibration of final model – Model B (CD4 included)

Derivation Dataset					Validation Dataset				
			Death					Death	
Decile	N	Cut off*	Observed**	Predicted†	Decile	N	Cut off*	Observed**	Predicted†
1	284	0.0026	0	0.6	1	272	0.0025	1	0.5
2	284	0.0036	0	0.9	2	271	0.0034	1	0.8
3	284	0.0046	0	1.2	3	272	0.0044	1	1.1
4	284	0.0059	1	1.5	4	271	0.0057	1	1.4
5	284	0.0084	3	2	5	272	0.0078	2	1.8
6	284	0.0123	1	2.8	6	271	0.0113	3	2.5
7	284	0.0193	5	4.4	7	272	0.0177	3	3.9
8	284	0.0325	7	7.1	8	271	0.0322	7	6.5
9	284	0.0704	15	13.4	9	272	0.068	14	12.7
10	284	0.807	52	50.1	10	271	0.7138	34	47.5
	2840		84	84		2715		67	79

Derivation dataset Hosmer-Lemeshow chi2(8) 5.21, p=0.7345
Validation dataset Hosmer Lemeshow chi2(8) = 5.73, p=0.677
* Upper boundary of predicted risk
**Observed = observed number dying within 6 months of ART initiation
†Predicted = expected number dying within 6 months of ART initiation
