# Supplementary figures and images for: Risk scores for predicting early antiretroviral therapy mortality in sub-Saharan Africa to inform who needs intensification of care: a derivation and external validation cohort study
Source: BMC Med. 2020 Nov 9;18:311. doi: 10.1186/s12916-020-01775-8 (PMC7650165; doi:10.1186/s12916-020-01775-8)

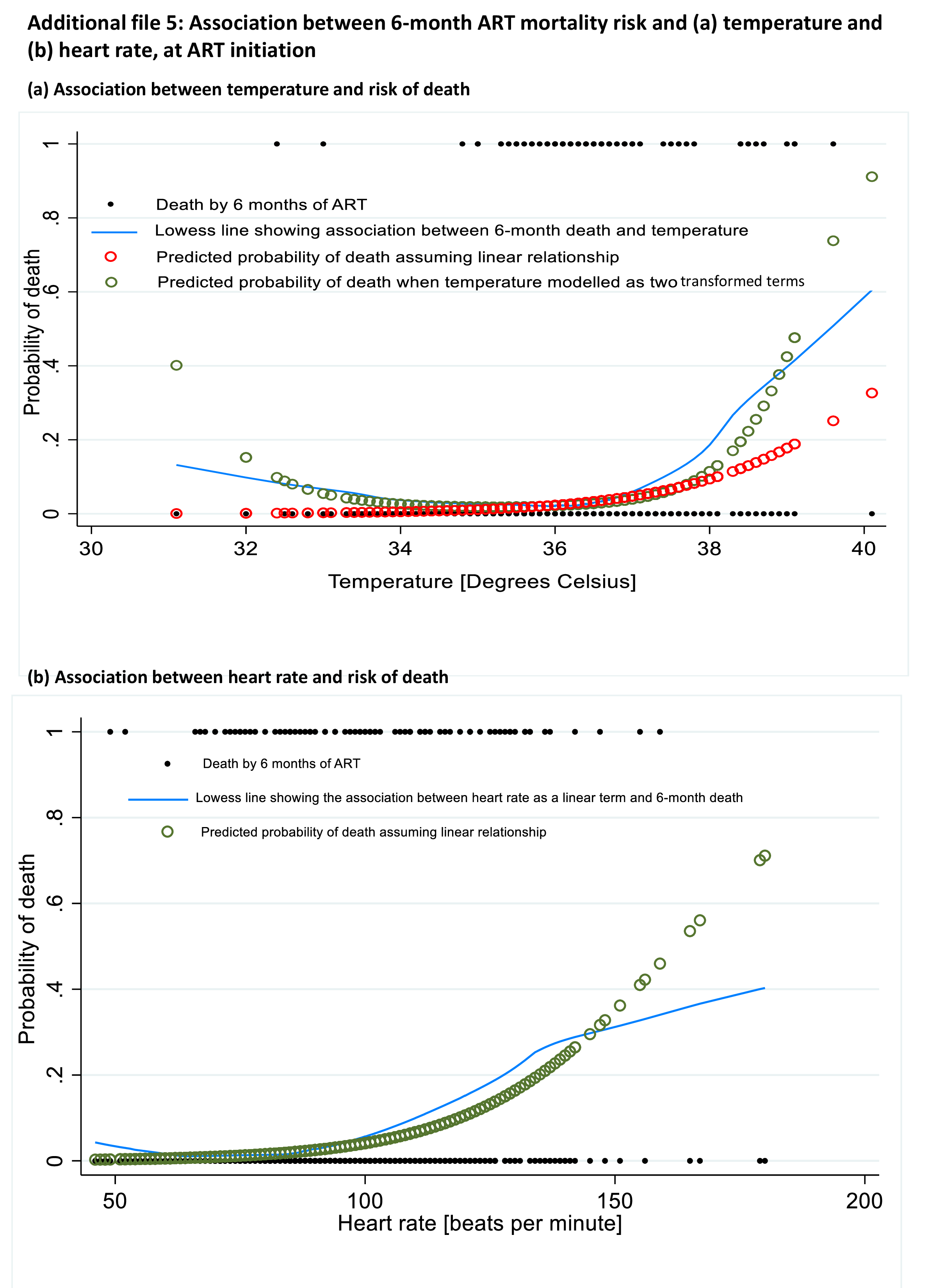

Supplement: Supplementary file 5 — Additional file 5. Figure showing association between 6-month ART mortality risk and (a) temperature and (b) heart rate, at ART initiation. [file 12916_2020_1775_MOESM5_ESM.tiff]

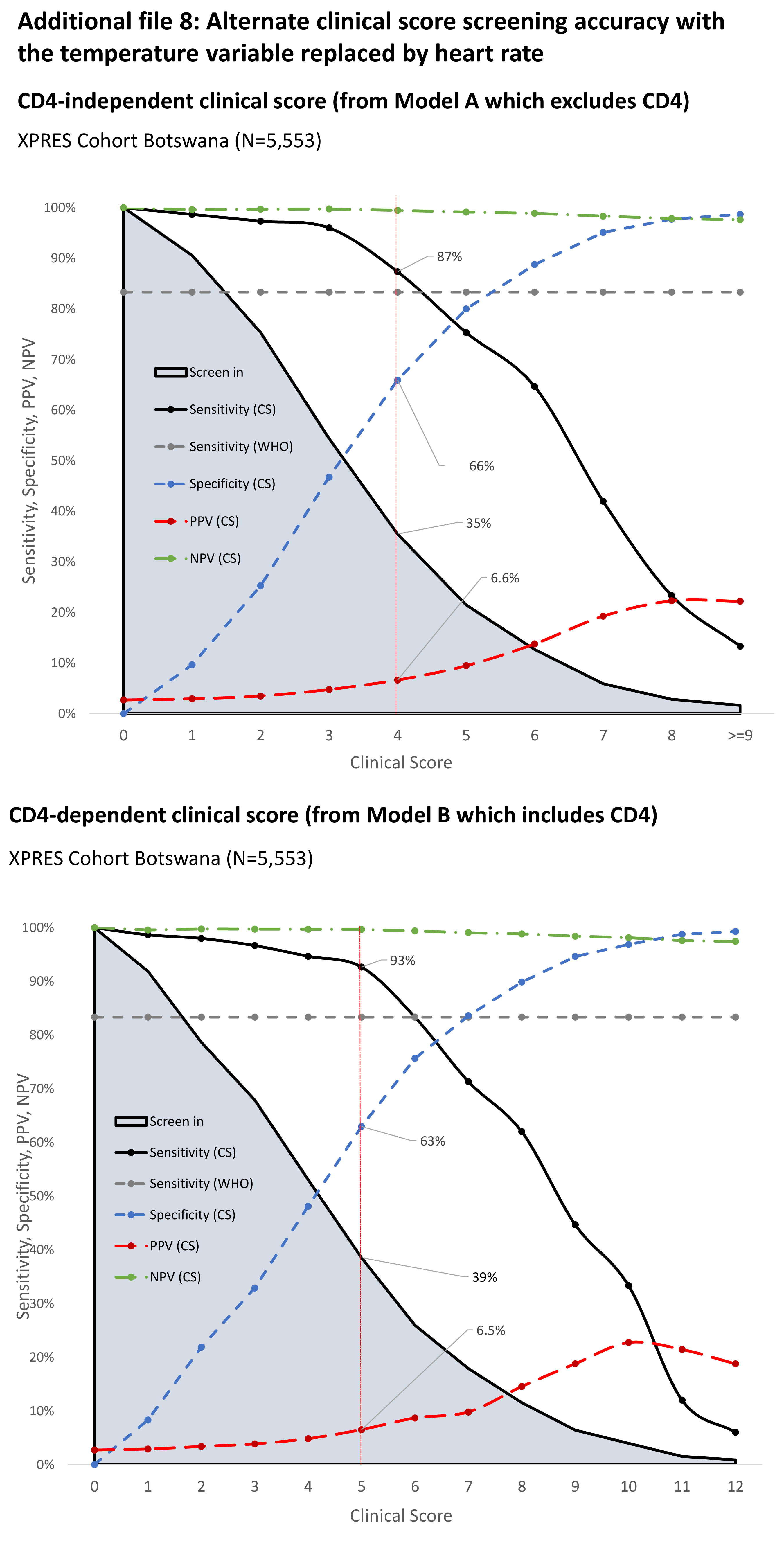

Supplement: Supplementary file 8 — Additional file 8. Figure showing alternate clinical score screening accuracy with the temperature variable replaced by heart rate. [file 12916_2020_1775_MOESM8_ESM.tiff]

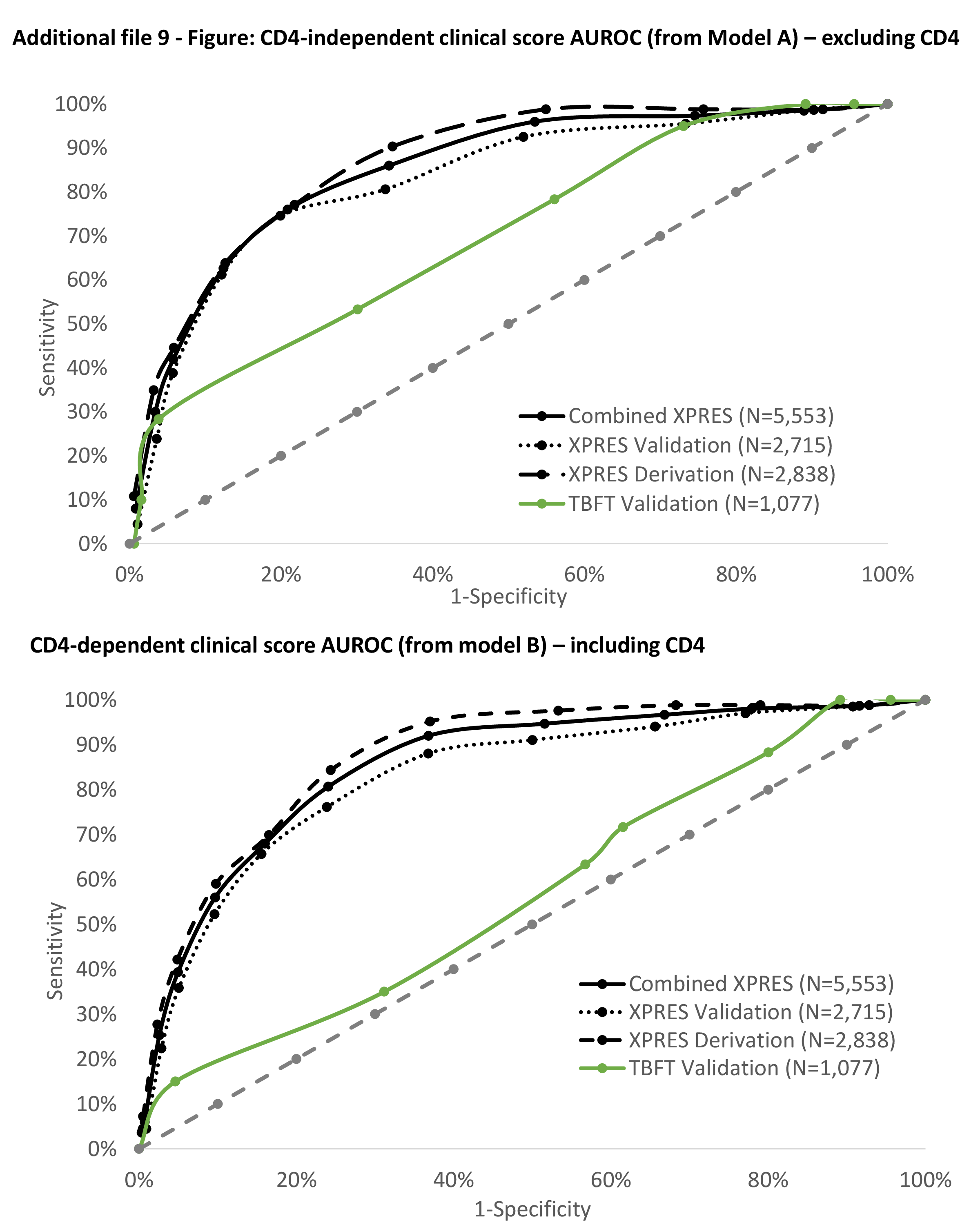

Supplement: Supplementary file 9 — Additional file 9. Figure showing area under the receiver operating characteristic curve for clinical score performance in combined XPRES dataset (N = 5553) and external validation TB Fast Track Dataset (N = 1077) for Models A (excluding CD4) and B (including CD4). [file 12916_2020_1775_MOESM9_ESM.tiff]
