# Supplementary material for: Risk scores for predicting early antiretroviral therapy mortality in sub-Saharan Africa to inform who needs intensification of care: a derivation and external validation cohort study
Source: BMC Med. 2020 Nov 9;18:311. doi: 10.1186/s12916-020-01775-8 (PMC7650165; doi:10.1186/s12916-020-01775-8)
Supplement: Supplementary file 6 — Additional file 6 Table showing multivariable model and clinical score generation from the derivation dataset with heart rate replacing the measured temperature variable (N = 2838). [file 12916_2020_1775_MOESM6_ESM.pdf]

Additional file 6: Multivariable model and clinical score generation from the derivation dataset with heart rate replacing the measured temperature variable (N = 2,838)

		Predictor - Model A (excluding CD4)					Predictor - Model B (including CD4)				
		**AOR**	**95% CI**	**p-value**	**ß coefficient**	**Score**	**AOR**	**95% CI**	**p-value**	**ß coefficient**	**Score**
Sex and pregnancy status	Female (pregnant)	1.00	--	--	--	0	1.00	--	--	--	0
	Female (non-pregnant)	1.93	(0.63-5.93)	0.251	0.66	1	1.72	(0.58-5.11)	0.328	0.54	1
	Male	4.11	(1.16-14.52)	0.028	1.41	2	3.50	(1.06-11.61)	0.041	1.25	2
Number of WHO TB symptoms	0	1.00	--	--		0	1.00	--	--	--	0
	≥1	3.55	(2.25-5.61)	<0.001	1.27	2	3.28	(2.06-5.23)	<0.001	1.19	2
WHO Stage	I/II	1.00	--	--		0	1.00	--	--	--	0
	III/IV	2.48	(1.26-4.87)	0.008	0.91	1	2.35	(1.19-4.65)	0.014	0.86	2
Heart rate at enrollment	≤120 beats/min	1.00	--	--		0	1.00	--	--	--	0
	>120 beats/min	6.63	(3.36-13.10)	<0.001	1.89	3	6.13	(3.25-11.54)	<0.001	1.81	3
CD4 count	≥200/µL					N/A	1.00	--	--	--	0
	<200/µL					N/A	1.84	(1.1-3.11)	0.021	0.61	1
Anemia Status[a]	No anemia	1.00	--	--		0	1.00	--	--	--	0
	mild/moderate anemia	4.77	(2.58-8.82)	<0.001	1.56	2	4.44	(2.45-8.05)	<0.001	1.49	3
	Severe anemia	10.03	(4.05-24.83)	<0.001	2.31	4	8.88	(3.71-21.24)	<0.001	2.18	4

Abbreviations: AOR, adjusted odds ratio; CI, confidence interval; WHO, World Health Organization.

[a]Anemia severity was classified according to World Health Organization criteria as follows: no anemia, hemoglobin level of ≥13.0 g/dL for men, ≥12.0 g/dL for non-pregnant females, and ≥11.0 g/dL for pregnant females; mild/moderate anemia, 8.0–<13.0 g/dL for men, 8.0–<12.0 g/dL for non-pregnant women, and 7.0-<11.0 g/dL for pregnant women; and severe anemia, <8.0 g/dL for males and non-pregnant females and <7.0 g/dL for pregnant women.
