# Supplementary material for: Risk scores for predicting early antiretroviral therapy mortality in sub-Saharan Africa to inform who needs intensification of care: a derivation and external validation cohort study
Source: BMC Med. 2020 Nov 9;18:311. doi: 10.1186/s12916-020-01775-8 (PMC7650165; doi:10.1186/s12916-020-01775-8)
Supplement: Supplementary file 7 — Additional file 7. Tables showing performance of clinical score in derivation and validation datasets for Models A (excluding CD4) and B (including CD4). [file 12916_2020_1775_MOESM7_ESM.pdf]

Additional file 7a: Performance of clinical score in derivation and validation datasets – Model A (excluding CD4)

	Derivation			Validation			TB Fast Track		
Clinical score	Total with score	Number died within 6 months	% Died	Total with score	Number died within 6 months	% Died	Total with score	Number died within 6 months	% Died
0	236	1	0%	293	1	0%	0	0	0
1	434	-	0%	415	2	0%	45	0	0%
2	573	-	0%	569	2	0%	65	0	0%
3	564	7	1%	490	8	2%	167	3	2%
4	367	11	3%	371	4	1%	183	10	5%
5	263	11	4%	214	9	4%	279	15	5%
6	203	16	11%	186	15	8%	282	15	5%
7	81	8	19%	66	10	15%	34	11	32%
8	92	20	38%	80	13	16%	16	6	38%
>=9*	25	9	27%	31	3	10%	6	0	0%
Total	2838	83		2715	67		1,077	60	

*In derivation dataset, 11, 11, and 3 patients had scores of 9, 10, and 11, with 6, 3, and 0 deaths respectively. In XPRES validation dataset, 14, 15, and 2 had scores of 9, 10, and 11 with 0, 2, and 1 deaths respectively. No scores >9 were observed in the TBFT dataset.

Additional file 7b: Performance of clinical score in derivation and validation datasets – Model B (including CD4)

	Derivation			Validation			TB Fast Track		
Clinical score	Total with score	Number died within 6 months	% Died	Total with score	Number died within 6 months	% Died	Total with score	Number died within 6 months	% Died
0	198	1	1%	257	1	0%	0	0	0
1	381	-	0%	361	1	0%	0	0	0
2	296	-	0%	305	2	1%	45	0	0%
3	413	1	0%	413	2	0%	65	0	0%
4	451	2	0%	351	2	1%	100	7	7%
5	358	9	3%	348	8	2%	198	10	5%
6	228	12	5%	226	7	3%	54	5	9%
7	195	9	5%	166	9	5%	277	17	6%
8	149	14	9%	131	11	8%	282	12	4%
9	82	12	15%	67	9	13%	56	9	16%
10	67	17	25%	63	12	19%			
11	8	3	38%	11	-	0%			
>=12	12	3	25%	16	3	19%			
Total	2838	83		2715	67		1,077	60	

*In derivation dataset, 9 and 3 patients had scores of 12 and 13, with 3, and 0 deaths respectively. In XPRES validation dataset, 14 and 2 had scores of 12 and 13 with 2 and 1 deaths respectively. No scores >9 were observed in the TBFT dataset.
